# Lighting up protein design

**DOI:** 10.7554/eLife.79310

**Published:** 2022-05-19

**Authors:** Grzegorz Kudla, Marcin Plech

**Affiliations:** 1 MRC Human Genetics Unit, The University of Edinburgh Edinburgh United Kingdom

**Keywords:** fitness landscape, molecular evolution, protein engineering, machine learning, GFP, *E. coli*

## Abstract

Using a neural network to predict how green fluorescent proteins respond to genetic mutations illuminates properties that could help design new proteins.

**Related research article** Gonzalez Somermeyer L, Fleiss A, Mishin AS, Bozhanova NG, Igolkina AA, Meiler J, Alaball Pujol M-E, Putintseva EV, Sarkisyan KS, Kondrashov FA. 2022. Heterogeneity of the GFP fitness landscape and data-driven protein design. *eLife* **11**:e75842. doi: 10.7554/eLife.75842.

Protein engineering is a growing area of research in which scientists use a variety of methods to design new proteins that can perform certain functions. For instance, enzymes that can biodegrade plastics, materials inspired by spider silk, or antibodies to neutralize viruses (Lu et al., 2022; Shan et al., 2022).

In the past, protein engineering has commonly relied on directed evolution, a laboratory procedure that mimics natural selection. This involves randomly mutating the genetic sequence of a naturally occurring protein to create multiple variants with slightly different amino acids. Various selection pressures are then applied to identify the ‘fittest’ variants that best carry out the desired role (Chen et al., 2018). Alternatively, researchers can use a rational design approach, in which new proteins are built using principles learned from the study of known protein structures (Anishchenko et al., 2021).

Now, in eLife, Fyodor A Kondrashov (from the Institute of Science and Technology Austria and the Okinawa Institute of Science and Technology Graduate University) and colleagues – including Louisa Gonzalez Somermeyer as first author – have combined elements of both approaches to engineer new variants of naturally occurring green fluorescent proteins (GFP; Gonzalez Somermeyer et al., 2022). First, the team (who are based at various institutes in Austria, Japan, the United States, the United Kingdom, Germany and Russia) generated tens of thousands of GFP variants that differed from each other by three to four mutations on average, and measured their fluorescence. This was used to create a ‘fitness landscape’ showing how the genetic sequence of each mutant relates to its performance (Figure 1). The data was then fed in to a neural network that can expand the landscape by predicting the performance of variants that were not observed experimentally.

**Figure 1.**
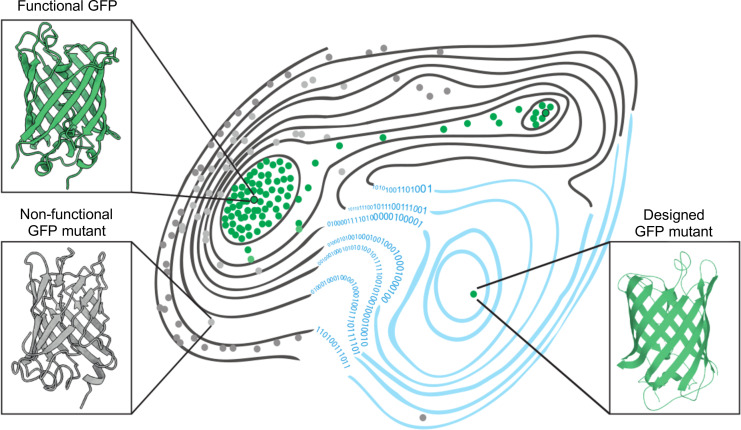
The fitness landscape of green fluorescent proteins. Fitness landscapes provide a graphical representation of how a protein’s genetic sequence relates to its performance, leading to a multidimensional surface made up of peaks, ridges, and valleys. In the fitness landscape shown, horizontal distance represents the number of mutations that separate variants of a protein, while vertical elevation represented by contour lines indicates the fluorescence of each mutant. Two naturally occurring green fluorescent proteins (GFPs; dots outlined in black) reside on different peaks of the landscape (top left and top right) and are connected by a narrow ridge (area of high fitness). Mutant proteins at the peaks and ridges are all functional and able to fluoresce (green dots), whereas those in the valleys are non-functioning (grey dots). Application of a machine learning algorithm expanded the fitness landscape (right; blue contour lines) by including mutations that are not generated by evolution. This led to the creation of functional, synthetic variants (green dot, bottom right) that reside on different fitness peaks to variants that are naturally occurring.

Image credit: Marcin Plech & Grzegorz Kudla (CC BY 4.0).

Using this machine learning approach, Gonzalez Somermeyer et al. were able to design fluorescent proteins that differed from their closest natural relative by as many as 48 mutations. This is remarkable because in most protein mutagenesis experiments it only takes a few mutations before the function of the protein deteriorates. Evolution, on the other hand, can generate functional variants that differ by hundreds of mutations through a process of trial and error, which is akin to walking along a narrow ridge of high fitness one mutational step at a time. The neural network, however, appears to have jumped straight to a distant peak of high fitness (Figure 1). So, how did the network know where to take a leap?

To answer this, Gonzalez Somermeyer et al. experimented with three GFP proteins that originated from evolutionarily distant species. They found that machine learning was better at generating functional variants of cgreGFP than its two homologues, amacGFP and ppluGFP2 (a fourth homologue, avGFP, was also studied, but not in the machine learning experiment). This allowed the team to look for properties within each protein’s genetic sequence and fitness landscape which correlated with its machine learning performance.

Analysis of the fitness landscape revealed that the homologues differed in the number of mutations they could tolerate: it took on average three to four mutations until the fluorescence of cgreGFP and avGFP deteriorated, but seven to eight mutations were needed to compromise the function of amacGFP and ppluGFP2. The proteins also differed in their general sturdiness: ppluGFP2 was stable when exposed to high temperatures, whereas the structure of cgreGFP was more sensitive to changes in temperature.

Finally, Gonzalez Somermeyer et al. found that the increased mutational sensitivity of avGFP and cgreGFP (and to a lesser degree ppluGFP2) was due to negative epistasis – that is, when an individual mutation is well tolerated, but has a negative effect on the protein’s function when combined with other mutations (Bershtein et al., 2006; Domingo et al., 2019). The reduced fluorescence of amacGFP, however, could be ascribed almost entirely to additive effects, with each mutation incrementally making the protein less functional.

In order to generate functional variants, the network needs an opportunity to learn which properties of the fitness landscape are relevant from the data provided. The findings of Gonzalez Somermeyer et al. suggest that to predict a protein’s function, the algorithm only requires data on the effects of single-site mutations and low-order epistasis (interactions between small sets of mutations). This is good news for the protein engineering field as it suggests that prior knowledge of high-order interactions between large sets of mutations is not needed for protein design. Furthermore, it explains why the neural network is better at generating new variants of cgreGFP, which has a sharp fitness peak and high prevalence of epistasis.

In sum, these experiments provide a successful case study in protein engineering. An interesting extension would be to analyse the three-dimensional structures of the variants using AlphaFold, an algorithm which can predict a protein’s structure based on its amino acid sequence (Jumper et al., 2021). This would reveal if data from AlphaFold improves the prediction of functional variants, and help to identify structural features that rendered some of the variants non-fluorescent despite them being predicted to work. In the near future, assessing a new variant’s structure before it is synthesized could become a standard validation step in the design of new proteins. Furthermore, studying the fitness landscapes of multiple related variants, as done by Gonzalez Somermeyer et al., could reveal how a protein’s genetic sequence and structure changed over the course of evolution (Hochberg and Thornton, 2017; Mascotti, 2022). A better understanding of the evolution of proteins will help scientists to engineer synthetic molecules that carry out specific roles.
